# Investigating the Relationship between Childbirth Type and Breastfeeding Pattern Based on the LATCH Scoring System in Breastfeeding Mothers

**DOI:** 10.1055/s-0041-1735985

**Published:** 2021-11-16

**Authors:** Tayebeh Mokhtari Sorkhani, Elahe Namazian, Samaneh Komsari, Shima Arab

**Affiliations:** 1 Department of Midwifery, School of Nursing and Midwifery, Bam University of Medical Sciences, Bam, Iran; 2 Zarand Social Security Hospital, Social Security Organization, Zarand, Kerman, Iran; 3 Research Department, Institute for Futures Studies in Health, Kerman University of Medical Sciences, Kerman, Iran; 4 School of Nursing and Midwifery, member of the Student Research Committee of Bam University of Medical Sciences, Bam, Iran, University of Medical Sciences, Bam, Iran

**Keywords:** cesarean section, natural childbirth, LATCH, breastfeeding, cesariana, parto natural, LATCH, amamentação

## Abstract

**Objective** The role of breast milk in the physical and mental health of infants and in the prevention of infant death is widely known. The benefits of breastfeeding for mothers and infants have been proven, but several factors can affect breastfeeding. Childbirth is one of the most influential factors. The present study aimed to investigate the effect of the type of delivery (natural childbirth and cesarean section) on breastfeeding based on the latch, audible swallowing, type of nipple, comfort, hold (LATCH) scoring system.

**Methods** The present cross-sectional observational study was performed using the census method among women who referred to Afzalipour Hospital for delivery in May 2020; the breastfeeding pattern was completed by observation and the in-case information, by LATCH checklist. Data were analyzed using the Statistical Package for the Social Sciences (IBM SPSS Statistics for Windows, IBM Corp., Armonk, NY, United States) software, version 19.0, analysis of variance (ANOVA), and the Chi-squared statistical test.

**Results** Out of a total of 254 deliveries (127 natural childbirths and 127 cesarean deliveries), there was no statistically significant difference between the 2 study groups in terms of age, maternal employment status, and infant weight, but there was a statistically significant relationship between the type of delivery, the maternal level of schooling, and the appearance, pulse, grimace, activity, and respiration (Apgar) score in the first minute. The mean score of breastfeeding patterns among the natural childbirth group (9.33) was higher than that of the cesarean section group (7.21).

**Conclusion** The type of delivery affects the mother's performance during breastfeeding, and mothers submitted to cesarean sections need more support and help in breastfeeding.

## Resumo

**Objetivo** É sabido o papel do leite materno na saúde física e mental dos bebês e na prevenção da mortalidade infantil. Os benefícios da amamentação para mães e bebês foram comprovados, mas vários fatores podem afetar a amamentação. O parto é um dos fatores mais influentes. Este estudo teve como objetivo investigar o efeito do tipo de parto (parto Natural e cesariana) na amamentação com base no sistema de pontuação agarramento, deglutição audível, tipo de mamilo, conforto, segurar ( *latch* , *audible swallowing* , *type of nipple* , *comfort* , *hold* , LATCH, em inglês).

**Métodos** Este estudo transversal e observacional foi realizado pelo método do censo entre mulheres que buscaram atendimento no Hospital Afzalipour para parto em maio de 2020; o padrão de amamentação foi completado por observação e in-case, pela lista de verificação do LATCH. Os dados foram analisados usando o programa Statistical Package for the Social Sciences (IBM SPSS for Windows, IBM Corp., Armonk, NY, Estados Unidos), versão 19.0, análise de variância ( *analysis of variance* , ANOVA, em inglês) e o teste estatístico do qui-quadrado.

**Resultados** De um total de 254 partos (127 parto naturais e 127 cesarianas), não houve diferença estatisticamente significativa entre os dois grupos de estudo em termos de idade, situação laboral materna e peso do bebê, mas houve uma relação estatisticamente significativa entre os tipo de parto, a escolaridade materna e o índice de aparência, frequência cardíaca, irritabilidade reflexa, tônus muscular, e respiração *appearance* , *pulse* , *grimace* , *activity* , *and respiration* , (Apgar), no primeiro minuto. A pontuação média do padrão de amamentação no grupo do parto natural (9,33) foi maior do que a do grupo da cesariana (7,21).

**Conclusão** O tipo de parto afeta desempenho da mãe durante a amamentação, e as mães submetidas a cesariana necessitam de mais apoio e ajuda na amamentação.

## Introduction

About 800 thousand infant deaths worldwide are due to the late onset of breastfeeding and the absence of exclusive breastfeeding. 1 One of the United Nations' Sustainable Development Goals (SDGs) is to reduce infant mortality by 12 per 1,000 live births, and deaths under the age of 5 to less than 25 per 1,000 live births by eliminating infant mortality by 2030. 2 Starting breastfeeding in the first hour after birth can reduce the risk of infant mortality by ∼ 45%, and infants who are exclusively breastfed are 14 times more likely to survive than those who are not breastfed. 3 Malnutrition is one of the leading causes of child mortality, and according to the United Nations Children's Fund (UNICEF, 2010), 4 ∼ 40% to 60% of children under the age of 5 years were affected by late-onset breastfeeding. According to various studies 3 and reports by the World Health Organization (WHO) and UNICEF, 4 5 breastfeeding is related to social, economic, demographic, behavioral, and cultural factors of mothers, the place and method of delivery, counseling and education about breastfeeding, and factors related to obstetric services. The method of delivery is one of the factors that affects breastfeeding. There is an increase in studies 3 6 examining the negative effects of cesarean section on well-being, maternal behavior, and breastfeeding physiology at the beginning of the postpartum period. To clarify this issue, it can be said that the anesthesia and painkillers used in cesarean sections have a negative effect on oxytocin secretion and milk production. Morphine has also been shown to stop breast milk secretion. 7 Cesarean delivery seems to have little effect on the mother-newborn relationship. 8 Women submitted to natural childbirth are 4.8 times more likely to continue breastfeeding exclusively for 30 days after delivery than women submitted to cesarean delivery. 9 Breastfeeding is associated with the early initiation of breastfeeding, and, as recommended in the steps of the Baby-Friendly Hospital Initiative (BFHI), breastfeeding should occur immediately after birth. 9 In a survey conducted in Saudi Arabia, that mothers who had a caesarean delivery had about 1.4 times higher odds for delaying Breast Feeding by >1 h when compared to mothers who had a vaginal delivery. 10 In one study, 11 the prevalence of late-onset breastfeeding in women submitted to natural childbirth was of 35.34% against 50.49% of women submitted to cesarean section, who were at a higher risk of performing non-exclusive breastfeeding during the 3 days after delivery. Cesarean section is a major abdominal surgery that can delay exclusive feeding. During the first few hours after a cesarean section, mothers are expected to begin caring for their baby at the same time as they are coping with postoperative problems, including pain. However, a cesarean section can have a negative effect on breastfeeding physiology, and can cause side effects that prevent the mother from being in contact with the newborn. Unbearable pain after the surgery and an increase in the newborn's need for intensive care negatively affect breastfeeding. 12

It has been shown that cesarean delivery is a risk factor for incomplete breastfeeding, and the rate of breastfeeding in the early hours after cesarean delivery is lower than that in the early hours after vaginal birth. 13 So far, the effect of childbirth on breastfeeding has been analyzed in countless studies, but no decisive result has been obtained. 14

Since breastfeeding is an important and effective factor in the growth of the newborn, is directly related to infant mortality, and is one of the indicators of global health, the present study aimed to investigate the pattern of breastfeeding among mothers submitted to natural childbirth and cesarean section to diagnose the issues regarding breastfeeding and their causes according to the type of delivery, to provide more counseling and supportive strategies to mothers to improve this process.

## Methods

### Study Design

The present cross-sectional observational study was conducted using the census method among women who referred to Afzalipour Hospital, in Kerman, in May 2020, for delivery. The women who underwent natural childbirth and cesarean section were included in the study regardless of previous pregnancy history and age. During the data collection, we excluded women who had undergone emergency cesarean section, those who had given birth preterm (under 34 weeks of gestational age) and whose newborns did not develop effective sucking power due to lack of proper coordination between sucking, swallowing and breathing, 15 and mothers whose newborns had been hospitalized or died in the neonatal ward. After delivery, the mothers received postpartum care performed by expert midwives and gynecologist professionals and were transferred to the Postpartum Surgery and Natural Childbirth Unit of the Department of Gynecology and Midwifery, , the researcher, who was trained in midwifery, went ot the same unit after obtaining the necessary permits and receiving the approval code from the Ethics in Reasearch Committee (IR.MUBAM.REC.1399.016). Then, the researcher explained to the mothers that participation in the research was completely voluntary. They were also assured that if they refused to participate in the research, they would not be deprived of diagnostic and therapeutic care, and even after agreeing to participate in the investigation, they could withdraw from it at any time, and would not be required to pay a fine or damages. They were informed that all of their information was kept confidential, and that the general and group results of the research would be published without mentioning the names and details of the participants. Then, the mothers' written consent was obtained through the questionnaire forms.

### Participant and Study Sampling

The sample size was calculated as 197 subjects based on similar studies 16 and a literature review. However, considering the period of the study (1 month), we decided to evaluate all available participants by census, and finally included 254 women.

The data collection tools were the latch, audible swallowing, type of nipple, comfort, hold (LATCH) Score for Breastfeeding Success Assessment and the personal data collection form, which included age, occupation, level of schooling, place of residence, number of children, gestational age, and previous breastfeeding history. The data collection form was filled out through questions and observation, as well as information contained in the medical records. The LATCH was used to assess breastfeeding patterns.

The LATCH was first published in 1994, and was used to identify the need for breastfeeding-related interventions. 17 18 The method of scoring is based on the appearance, pulse, grimace, activity, and respiration (Apgar) score, and results range from 0 to 10. A score < 10 indicates the mother's need for more support during breastfeeding. The system consists of five components, including how the baby latches, audible swallowing, the type of nipple, the mother's level of comfort (regarding the breast and nipple), and the level of assistance needed by the mother in order to hold the newborn while breastfeeding; the scores for each component can be 0 (weak), 1 (relatively good), or two (good).

The validity and reliability of this tool have been studied and confirmed in several studies. 16 17 18 19 In a 2011 study by Karimi et al., 16 the Cronbach α coefficient was of 0.71.

The study data were entered into the Statistical Package for the Social Sciences (IBM SPSS Statistics for Windows, IBM Corp., Armonk, NY, United States) software, version 19.0, and descriptive and analytical statistics were used to analyze the results and the correlations among the variables.

### Ethical Consideration

The present article is the result of a dissertation approved by the Bam University of Medical Sciences under the code of ethics number (IR.MUBAM.REC.1399.016) and supported by the university's research deputy. The purpose of the study was explained to the subjects, and they were included after signing the written informed consent.

### Statistical Analysis

The data were analyzed using the SPSS version 19.0. Descriptive statistics (frequency, percentage, mean, and standard deviation) were used to describe the characteristics of the sample. The Chi-squared test and the parametric equivalent (analysis of variance, ANOVA) was also used. *P* value less than 0.05 was considered as statistically significant.

## Results

The sample of the present study was compsed of 254 participants: 127 women submitted to natural childbirth, and 127 who underwent cesarean section. The mean age of the sample was 27.5 ± 2.3 years. The weight of the newborns in both groups ranged from 1,100 g to 5,200 g. The mean newborn weight in the natural childbirth group was of 3,021 g, and, in the cesarean section group, of 2,855 g, which was not statistically significant ( *p* = 0.38). The majority of the participants were housewives. In terms of the level of schooling, most of the participants in Natural Childbirth group had not graduated from high school, and, in the cesarean section group, most participants had graduated from high school. Statistically, there was no difference between the two groups in terms of employment status, place of residence, newborn gender, mean age of mothers, and newborn weight. In both groups, the average number of previous children was two. In total, 26% of the newborns examined had been born preterm (≥ 34 weeks of gestational age) by cesarean section. However, in the natural childbirth group, only 13.4% of infants were preterm ( *p* = 0.006) ( Table 1 ).

**Table 1:** Comparison of employment status, place of residence, newborn gender and time of birth according to the type of delivery

		Cesarean section: *n* (%)	Natural childbirth: *n* (%)	*p* -value*
Employment status	Housewife	119 (93.7)	125 (98.4)	0.05
	Employed	8 (6.3)	2 (1.6)	
Place of residence	Urban center	89 (70.1)	78 (61.4)	0.14
	Countryside	38 (29.9)	49 (38.6)	
Newborn gender	Girl	66 (52)	58 (45.7)	0.19
	Boy	61 (48)	69 (54.3)	
Time of birth	Term	93 (73.2)	110 (86.6)	0.006
	Preterm	34 (26.8)	17 (13.4)	
Total		127 (100)	127 (100)	

Note: *Chi-squared test.

The results of the statistical tests show that the type of delivery was related to the mother's level of schooling, the rate of cesarean delivery was higher in mothers with a high school degree ( *p* = 0.03) ( Table 2 ).

**Table 2:** Comparison of the mother's level of schooling and the type of delivery

		Cesarean section: *n* (%)	Natural childbirth: *n* (%)	*p* -value*
**Level of schooling**	Illiterate	12 (9.4)	13 (10.2)	0.03
	Incomplete high school	42 (33)	61 (48.0)	
	Complete high school	48 (37.8)	44 (34.6)	
	Associate degree	6 (4.7)	2 (1.6)	
	Bachelor's degree	18 (14.2)	6 (4.7)	
	Master's degree or higher	1 (0.8)	1 (0.8)	
**Total**		127 (100)	127 (100)	

Note: *Chi-squared test.

For 99.2% of the natural childbrith group and 95.9% of the cesarean section group, the Apgar score in the first minute was ≥ 9 ( *p* = 0.03). However, regarding the Apgar score in the fifth minute, no statistically significant differences between the two groups were found ( *p* = 0.13) ( Table 3 ).

**Table 3:** Comparison of the Apgar scores and the type of delivery

		Cesarean section: *n* (%)	Natural childbirth: *n* (%)	*p* -value*
1-minute Apgar score	7	5 (3.9)	1 (0.7)	0.03
	8	16 (12.5)	9 (7.09)	
	9	106 (83.4)	117 (92.2)	
	10	0	0	
Total		127 (100)	127 (100)	
5-minute Apgar score	7	0	0	0.03
	8	2 (1.6)	1 (0.8)	
	9	6 (4.7)	1 (0.8)	
	10	119 (93.7)	125 (98.4)	
Total		127 (100)	127 (100)	

Abbreviation: Apgar, appearance, pulse, grimace, activity, and respiration.

Note: *Chi-squared test.

In total, 52.8% of the natural childbirth group and 53.5% of the cesarean section group had previous breastfeeding experience, but no statistically significant relationships were found between that and the type of delivery ( *p* = 0.52) ( Table 4 ).

**Table 4:** Comparison of previous breastfeeding experience and the type of delivery

		Cesarean section: *n* (%)	Natural childbirth: *n* (%)	*p* -value*
Previous breastfeeding experience	Yes	68 (53.5)	67 (52.8)	0.52
	No	59 (46.5)	60 (47.8)	
Total		127 (100)	127 (100)	

Note: *Chi-squared test.

An examination of the breastfeeding pattern and a Comparison of breastfeeding pattern score based on LATCH scores in both groups revealed statistically significant differences in all subsets of the LATCH score, with the mean score of the natural childbirth group (9.33) being higher than that of the cesarean section (7.21) group ( *p* ≤ 0.0001) ( Table 5 ).

**Table 5:** Comparison of LATCH scores and the type of delivery

LATCH	Cesarean section: mean ± standard deviation	Natural childbirth: mean ± standard deviation	*p* -value *
Latch	1.54 ± 0.67	1.75 ± 0.54	0.009
Audible nwallowing	0.61 ± 0.73	1.69 ± 0.55	< 0.0001
Type of Nipple	1.83 ± 0.43	1.94 ± 0.22	0.012
Comfort (breast/nipple)	1.94 ± 0.22	1.94 ± 0.12	0.009
Hold (positioning)	1.28 ± 0.76	1.96 ± 0.13	< 0.0001
Total (average score)	7.21	9.33	< 0.0001

Abbreviation: LATCH, latch, audible swallowing, type of nipple, comfort, hold.

Note:*Analysis of variance (ANOVA).

## Discussion

As shown by the findings of the present study, the type of delivery was directly related to the mother's level of schooling. The rate of cesarean delivery was higher in mothers with a high school degree. In the study by Ahmad-Nia et al. (2009), 20 the level of schooling was higher among mothers in the cesarean section group. In the studies by Hassanzadeh et al. (2019), 21 Valiani and Heshmat (2018), 22 and Islami et al. (2008), 23 the cesarean section group had complete secondary education, which is in line with with the present study. 21 22 23 Perhaps the reason for the lower level of schooling of the mothers in the present studyis the referral of people with with low education and therefore average income, to the Afzalipour Hospital and the use of free and low-cost insurance and childbirth services in this center compared with private and expensive medical centers.

In the present study, the Apgar score among the natural childbirth group was higher than that of the cesarean section group, which is consistent with the results of the study by Raafati et al. (2006). 24 However, in the study by Jafari et al. (2016), 25 there was no statistically significant relationship between the Apgar score and the type of delivery. During natural childbirth, it is easier to breathe because of the outflow of fluid from the lung of the fetus due to the pressure exerted by labor contractions and the passage of the fetus through the birth canal, and the Apgar score is expected to be higher. 26

In the present study, the number of preterm newborns delivered by cesarean section was higher than the number of those delivered by natural childbirth, which is in line with the studies by Namakin et al. (2011) 27 and Goldenberg et al. (2008). 28 However, this result is not consistent with the study by Maroufizadeh et al. (2016), 29 in which the authors mention that there was no separation between emergency and elective cesarean sections, on the other hand their study was performed in Tehran, where treatment status and healthcare facilities are better than in other provinces. In the present study, due to the referral of Afzalipour Medical Center, perhaps one of the reasons for the increase in preterm infant statistics in cesarean delivery is the occurrence of maternal and fetal problems that lead to rapid termination of labor and increase in preterm infants.

In the present study, the mean total LATCH score of the natural childbirth group was 9.33, and that of the cesarean section group was 7.21, and this difference was statistically significant. It should be noted that the mentioned scores were not related to pregnancy ranking and breastfeeding experience.

In the study by Parsay et al. (2005), 30 although the mean duration of breastfeeding in the natural childbirth was shorter than that of the cesarean section group, the breastfeeding pattern did not different much between the groups. In the study by Islami et al. (2008), 23 breastfeeding during the first hour of birth was higher among the natural childbirth group than among the cesarean section group, which was statistically significant. Karimi et al. (2011) 16 observed a significant difference in breastfeeding patterns between the natural childbirth and cesarean section groups. Cakmak and Kuguoglu (2007) 31 compared the breastfeeding pattern after both types of deliveries based on the LATCH score, and found a statistically significant difference. They also showed that the lower LATCH scores among mothers in the cesarean section group indicated that these individuals needed more support during hospitalization and at the beginning of breastfeeding, especially in terms of holding the baby. 31 A study conducted in Turkey by Gungor et al. (2004) 32 also confirmed that mothers submitted to cesarean section experienced several problems such as fatigue, insomnia, and breastfeeding problems that affected both their recovery process and neonatal care. In a more detailed study, 33 the mean score of how to breastfeed a baby in the vaginal delivery group was higher than in the cesarean section, and the position of the nipple, significantly better, in the natural childbirth group than in the cesarean section group. This can be due to the mother's general physical status for breastfeeding, which tends to be better after natural childbirth. The anesthesia and analgesics used in cesarean sections, such as morphine sulfate, can affect the secretion of oxytocin and stop the secretion of milk from the breast. 33 Intravenous injection of narcotics during cesarean delivery affects the newborn's normal reflexes in the first hours of life, and reduces the likelihood of the onset of breastfeeding. 34 However, when natural childbirth occurs spontaneously, without the use of tools and drugs, and the newborn is immediately exposed to the mother's skin, the chances that breastfeeding will begin then increase. 35

A study conducted in Tehran by Ekhtiari and Emami (2008) 36 showed that breastfeeding until the newborn is 3 months old is significantly more likely to occur among mothers in the natural childbirth group. Newborns delivered by cesarean section experience a delay in breastfeeding due to maternal problems. 37 In a study conducted in the United States by Evans et al. (2003), 7 the authors found that cesarean delivery reduced the rates of success in breastfeeding. The reasons were the mother's despair about unplanned and emergency cesarean section, the longer recovery time, higher levels of pain, and the greater risks posed by cesarean delivery. During the first few weeks after delivery, mothers submitted to cesarean sections should be taught about proper breastfeeding. Increasing the rate of exclusive breastfeeding requires more support and assistance to mothers submitted to cesarean sections, especially to create the right conditions for the mother to sit and hug the newborn to begin breastfeeding. 38

According to Morton et al., 39 “The increased rate of cesarean delivery is mainly attributable to a greater number of procedures performed for slow progress in labor, breech presentation or repeat cesarean section.” Puhl et al. 40 reported that induction of labor at 34 weeks of gestation is often linked to an increased risk of cesarean section. Byerly et al. (2020) 41 showed no independent association between prematurity and the likelihood of the onset of breastfeeding right after birth. Cesarean section is associated with in-hospital formula feeding and a higher proportion of breastfeeding difficulties. 42 The World Health Organization (WHO) 43 recommends that health education for women is an essential component of antenatal care, implementation of evidence-based clinical practice guidelines with timely second opinion and collaborative midwifery-obstetrician model of care. A model of supporting based on care provided primarily by midwives. 43 We also believe that mothers need to be educated by health care providers to select natural childbirth for improving their health and the health of their newborns.

## Conclusion

Natural childbirth is associated with a better maternal performance during breastfeeding, and mothers submitted to cesarean sections need more support and assistance in breastfeeding. Moreover, mothers should be encouraged to choose natural childbirth and start and continue breastfeeding.
